# Antimicrobial and Antibiofilm Activity of Curcumin-Loaded Electrospun Nanofibers for the Prevention of the Biofilm-Associated Infections

**DOI:** 10.3390/molecules26164866

**Published:** 2021-08-11

**Authors:** Anna Di Salle, Gianluca Viscusi, Francesca Di Cristo, Anna Valentino, Giuliana Gorrasi, Elena Lamberti, Vittoria Vittoria, Anna Calarco, Gianfranco Peluso

**Affiliations:** 1 Research Institute of Terrestrial Ecosystems (IRET)—CNR, Via Castellino, 111, 80131 Naples, Italy; anna.disalle@cnr.it (A.D.S.); anna.valentino@uniupo.it (A.V.); gianfranco.peluso@cnr.it (G.P.); 2 Department of Industrial Engineering, University of Salerno, Via Giovanni Paolo II 132, 84084 Fisciano, Italy; gviscusi@unisa.it; 3 Elleva Pharma s.r.l., Via Pietro Castellino 111, 80131 Naples, Italy; francesca.dicristo@ellevapharma.com; 4 Department of Pharmaceutical Sciences, Università del Piemonte Orientale “A. Avogadro”, Largo Donegani, 2, 28100 Novara, Italy; 5 Nice Filler s.r.l., Via Loggia dei Pisani, 25, 80133 Naples, Italy; elena.lamberti@nicefiller.it (E.L.); vvittoria@unisa.it (V.V.)

**Keywords:** electrospinning, drug delivery, biomedical applications, controlled release kinetics, curcumin

## Abstract

Curcumin extracted from the rhizome of *Curcuma Longa* has been used in therapeutic preparations for centuries in different parts of the world. However, its bioactivity is limited by chemical instability, water insolubility, low bioavailability, and extensive metabolism. In this study, the coaxial electrospinning technique was used to produce both poly (ε-caprolactone) (PCL)–curcumin and core–shell nanofibers composed of PCL and curcumin in the core and poly (lactic acid) (PLA) in the shell. Morphology and physical properties, as well as the release of curcumin were studied and compared with neat PCL, showing the formation of randomly oriented, defect-free cylindrical fibers with a narrow distribution of the dimensions. The antibacterial and antibiofilm potential, including the capacity to interfere with the quorum-sensing mechanism, was evaluated on *Pseudomonas aeruginosa* PAO1, and *Streptococcus mutans*, two opportunistic pathogenic bacteria frequently associated with infections. The reported results demonstrated the ability of the Curcumin-loading membranes to inhibit both PAO1 and *S. mutans* biofilm growth and activity, thus representing a promising solution for the prevention of biofilm-associated infections. Moreover, the high biocompatibility and the ability to control the oxidative stress of damaged tissue, make the synthesized membranes useful as scaffolds in tissue engineering regeneration, helping to accelerate the healing process.

## 1. Introduction

Among natural compounds, curcumin (1,7-bis(4-hydroxy-3-methoxyphenyl)-1,6-heptadiene-3,5-dione), the main active components isolated from the rhizome of *Curcuma Longa* L., is well known for its beneficial effect on human health because of its outstanding anti-inflammatory [1], antioxidant [2], anticancer [3], wound healing [4,5], and antibacterial [6] properties. Despite these health-beneficial effects, curcumin exhibits poor bioavailability, photodegradation, and in vivo instability (i.e., low serum levels, limited tissue distribution, excessive metabolism, etc.) that limits its therapeutic efficacy. To date, there is a large body of published studies supporting the use of several nanoformulations to overcome these limitations [7,8,9].

In particular, electrospinning (ES) has emerged as a versatile technique for preparing nano and microfibers of natural and synthetic polymers. The electrospun fibers show fitness in diversified technological fields such as filtration, protective clothing, wound healing, and other biomedical applications [10,11,12,13]. In particular, the use of electrospun fibers is highly promising for scaffolds in tissue engineering and drug delivery [13,14,15,16,17]. High surface area, high drug loading capacity, porosity, simultaneous delivery of different therapeutic agents, adequate mechanical strength, and cost-effectiveness are appealing characteristics for use in drug delivery systems [18,19,20,21,22,23,24,25,26,27]. In this context, the coaxial electrospinning technique, combining the properties from two different materials into a single core-sheath fiber, provides important and unique features relevant for biomedical applications [28,29,30,31,32,33,34].

The core–shell methodology might contribute to overcoming some challenges associated with electrospinning techniques (i.e., blending electrospinning, production of monolithic fibers, bilayer electrospinning) such as the incorporation of high compound loading, preservation of the activity and presence of burst effect. The use of core–shell structures allows for better tuning of the release kinetics by tailoring the thickness of shell polymer, which acts as a diffusion barrier for the compound loaded in the core as well as a protection of the active molecule from the sunlight or from microbiological attack for example. Then, coaxial fibers allow for the bioactive molecule to be located either in the core or shell layer in a simple one-step process and to modulate the thickness of fibrous material triggering the drug release kinetic [10]. Moreover, the drug release mechanism is mostly explained by the relative rates of erosion and diffusion of the entire fiber scaffold, hence the composition and the porosity of the single fibers can both modify this effect [35,36,37,38].

For these reasons, recently electrospinning technique was used in the treatment and prevention of biofilm-related infections, where the possibility to finely trigger the drug release may have a different impact on the bacterial/biofilm growth [39,40,41]. Biofilm is a complex multidimensional, self-sustained community of bacteria producing a matrix consisting of proteins, extracellular DNA, and polysaccharides, which are frequently associated with antibiotic resistance. Recently, several studies reported the use of electrospun fibers loaded with curcumin which ensure a controlled and sustained drug release in biomedical applications [5,42,43]. However, only a few papers described the fabrication and the application of coaxial electrospun mats loading curcumin [44,45]. Here we report the synthesis of coaxial membranes with two biodegradable polymers, poly (ε-caprolactone) (PCL) and poly (lactic acid) (PLA), used for the core and shell and loaded with curcumin in the core. The antibacterial and antibiofilm potential was evaluated on *Pseudomonas aeruginosa* PAO1, and *Streptococcus mutans*, two opportunistic pathogenic bacteria frequently associated with the infection of biomedical devices both in oral and in orthopedic implantology [46,47,48]. The Gram-negative bacterium PAO1 is responsible for several medical device-related infections (i.e., endocardial valve infection, ventilator-associated pneumonia, catheter-associated urinary tract infections, etc.), causing approximately 80% of severe infections in immunocompromised patients, of which 25–60% fatal [46,49,50]. *Streptococcus mutans*, moreover, is a Gram-positive bacterium present in the oral cavity and is one of the major contributors to dental biofilms [51,52].

The results reported herein demonstrate the ability of the single-needle-spinned PCL-Cur and the coaxial membrane PCL-Cur/PLA (PCL in the core and PLA in the shell) to inhibit both PAO1 and *S. mutans* biofilm growth and activity. The high biocompatibility and the ability to control the oxidative stress of damaged tissue, make the synthesized membranes useful as scaffolds in tissue engineering regeneration, helping to accelerate the healing process and to prevent biofilm-associated infections.

## 2. Results and Discussion

### 2.1. Morphological Analysis

**Figure 1:**
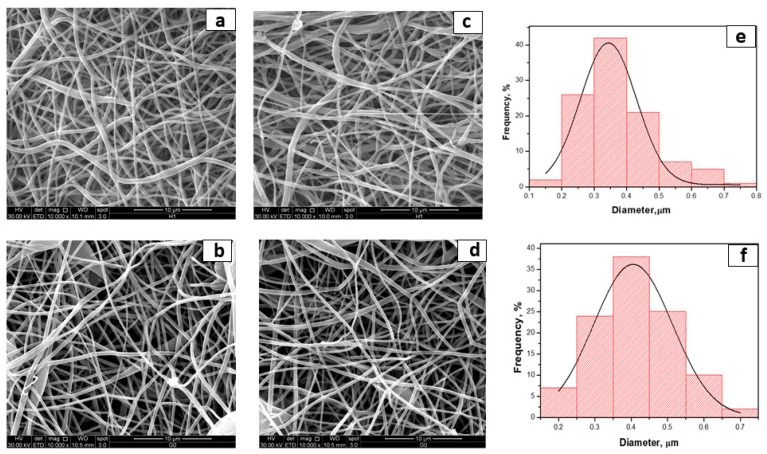
SEM photographs of (**a**) PCL; (**b**) PCL-PLA; (**c**) PCL-Cur; (**d**) PCL-Cur/PLA; (**e**) fiber distribution of PCL-Cur and (**f**) fiber distribution of PCL-Cur/PLA.

Electrospinning conditions were optimized to produce fibrous mats with bead-less fibers [53,54]. By adopting the chosen final parameters (as reported in the experimental part), fibers loaded with curcumin were successfully fabricated. Representative SEM photographs and the fiber diameter distribution of the electrospun membranes are shown in Figure 1.

**Figure 2:**
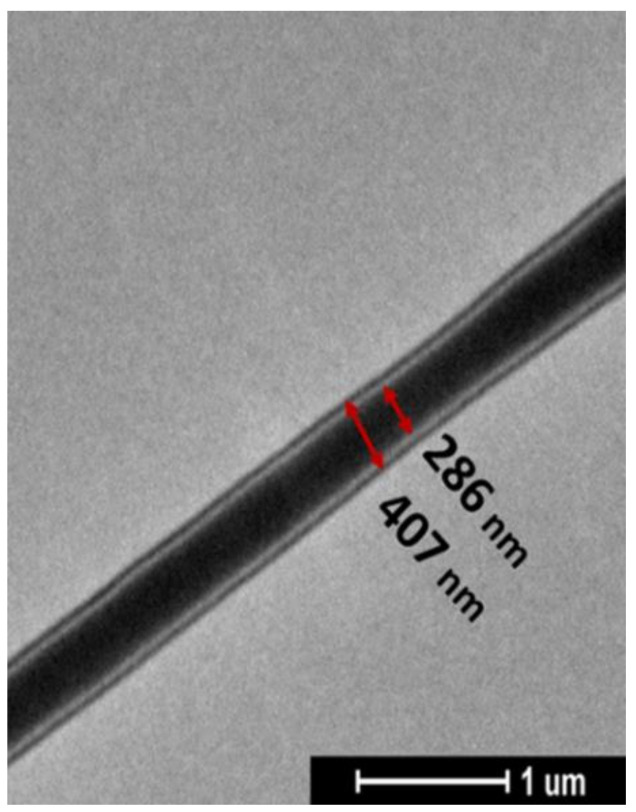
TEM images of PCL-Cur/PLA fiber.

As shown in Figure 1; curcumin loading did not noticeably affect the fiber morphology. The electrospinning of PCL led to the defection of free fibers, with a narrow fiber diameter distribution of 344 nm. Even in the case of coaxial fibers, the fibers are well-formed and almost free of defects. Their dimensions are slightly smaller than the neat PCL membrane and are very similar. In Figure 2, the TEM analysis shows the morphology of coaxial nanofibers where the inner and outer diameters made of PCL-Cur and PLA, respectively, are clearly evident.

It is evident that the core containing the drug is thicker than the shell of PLA; the PCL core is 286 nm, whereas the PLA shell is 60.5 nm.

### 2.2. Thermogravimetric Analysis

**Figure 3:**
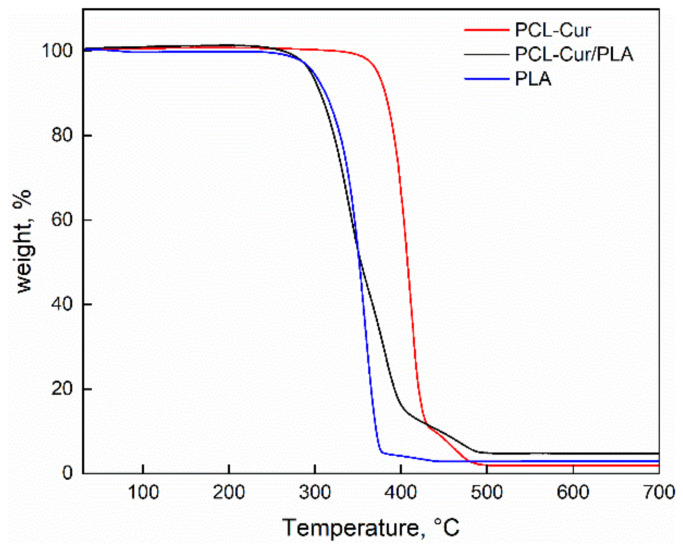
Thermogravimetric analysis of PCL-Cur, PCL-Cur/PLA and PLA.

Thermogravimetric analysis was performed for investigating the degradation temperature of the coaxial fibers compared to the pure polymers. The thermal decomposition up to 700 °C in the air of the electrospun fibers, either PCL-Cur or coaxial PCL-Cur/PLA, are shown in Figure 3.

The degradation behavior of the coaxial membrane is higher than the PCL-Cur. This is due to the presence of PLA that shows lower thermal stability. Table 1 reports the degradation temperatures as the onset and endset of the coaxial fibers compared to the pure polymers.

### 2.3. Mechanical Characterization

The mechanical properties of PCL-Cur membranes were evaluated from stress–strain curves and compared to the coaxial system. The mechanical parameters are reported in Table 2.

Interestingly, the coaxial fibers show a higher mechanical modulus due to the strengthening of one material inside the other. At variance, the coaxial systems displayed a lower extensibility than PCL system as well as reduced stress at the breakpoint. This can be due to the dishomogeneity of the systems. However, the decrease of these parameters is not dramatic, and does not compromise the mechanical properties, also considering the increase of the elastic modulus.

### 2.4. Drug Release Analysis

**Figure 4:**
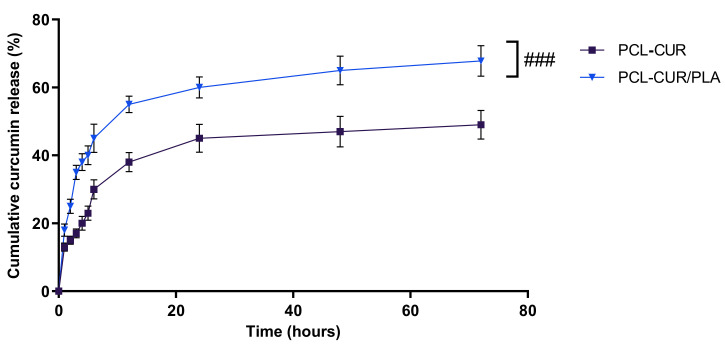
Cumulative curcumin release kinetic release profiles at 37 °C of PCL, and PCL-Cur/PLA incubated for 72 h in release medium (PBS/EtOH 90:10 *v/v*). For each sample, six different experiments were conducted, and the results expressed as the mean of the values obtained (mean ± SD). Statistically significant variations: ^###^ *p* < 0.001 versus PCL-CUR.

As curcumin is poorly soluble in aqueous solutions, the release kinetic was determined in PBS/EtOH (90:10 *v/v*), a medium that facilitates the solubilization of the curcumin. As shown in Figure 4, PCL-Cur/PLA exhibited a significantly (*p* < 0.001) more sustained curcumin release, compared to PCL-Cur, after 2 h of incubation and throughout the experimental period.

Approximately 30 and 45% of the curcumin was released within the first 6 h from PCL-Cur and PCL-Cur/PLA, respectively, indicating that curcumin molecules weakly attached on the fiber surfaces have a high diffusion tendency. This initial burst release is followed by a slower and constant release, reaching 49 and 67.8% of a curcumin release after 72 h of incubation for PCL-Cur and PCL-Cur/PLA, respectively. From 72 h onwards, the release of curcumin reached a plateau, leveling off at around 52 and 69% for PCL-Cur and PCL-Cur/PLA, respectively (data not shown).

Moreover, the coaxial membrane PCL-Cur/PLA showed a higher curcumin loading efficiency in comparison to the PCL-Cur membrane, incorporating 70 ± 3% and 56 ± 2% of curcumin, respectively. The obtained data are in accordance with the literature, confirming the capacity of the coaxial spinning to load the drug and control the initial burst release more efficiently with respect to single-needle spinning [55,56]. Furthermore, the incorporation into the core–shell of the fiber is helpful to protect unstable active molecules from degradation, serving as a physical barrier [32,56].

### 2.5. Antibacterial and Antibiofilm Analyses

**Figure 5:**
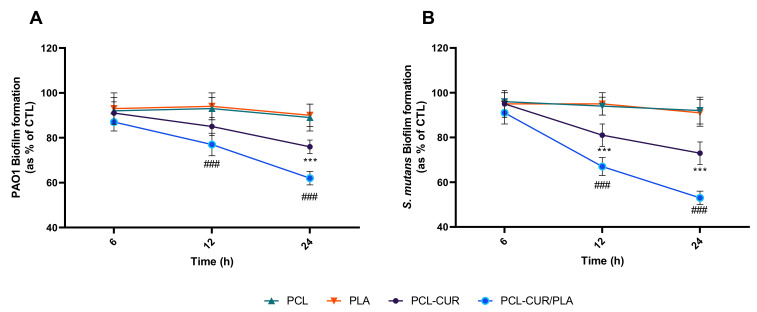
Antibiofilm activity of curcumin-loading membranes. Biofilm formation was evaluated by CV assay, after 6, 12, and 24 h of incubation at 37 °C in the presence of PAO1 (**A**), and *Streptococcus mutans* (**B**) as described in the material and methods section. Biofilm formation was reported as a percentage in comparison to the maximum amount of biofilm produced by PAO1 and *Streptococcus mutans* grown (bacterial positive controls). For each sample, six different experiments were conducted, and the results expressed as the mean of the values obtained (mean ± SD). Statistically significant variations: *** *p* < 0.001 versus PCL and PLA; ^###^ *p* < 0.001 versus PCL-Cur.

Biofilm inhibition was analyzed at different times, as reported in Figure 5.

The results indicated a significant decrease (*p* < 0.001) in both PAO1 and *S. mutans* biofilm formation only for the PCL-Cur and PCL-Cur/PLA, according to the release study.

In particular, the major effect was observed for PCL-Cur/PLA membrane whit a reduction of 38 ± 3%, 47 ± 3% in PAO1 and *S. mutans* biofilm formation, respectively. During the first stage of biofilm formation, only PCL-Cur/PLA significantly inhibited biofilm formation, resulting in a reduction of 23 ± 5%, 33 ± 4% in PAO1 and *S. mutans* biofilm formation, respectively after 12 h of incubation.

Moreover, to better understand the mechanism of action of curcumin released from membranes in the inhibition of biofilm formation/maturation, the mRNA level of several genes involved in the quorum-sensing process was evaluated by q-PCR.

In general, QS is regulated by different autoinducer molecules in Gram-negative and Gram-positive bacteria [57]. Indeed, Gram-negative bacteria such as PAO1 during the QS process produce extracellular autoinducers such as *N*-(3-oxododecanoyl)-l-homoserine lactone (3-*O*-C12-HSL) and *N*-butyryl-l-homoserine lactone (C4-HSL) that promote the transcription of virulence genes (i.e., exotoxin A, proteases, and rhamnolipids) [58,59]. Among them, rhamnolipids, which are of great importance acting as heat-stable extracellular hemolysins [60], are synthesized via *rhlAB* operon [61]. Conversely, Gram-positive bacteria such as *S. mutans* regulated the production of virulence factors via a two-component signal transduction system (TCSTS), consisting of a membrane-bound histidine kinase (HK) sensor protein, and a cognate cytoplasmic response regulator (RR) protein [62]. In *S. mutans*, the TCSTS consist principally in the operon ComAB/ComCDE where comC gene encodes competence-stimulating peptide (CSP), comD encodes HK sensor protein (ComD), and comE encodes an RR protein (ComE) [63].

**Figure 6:**
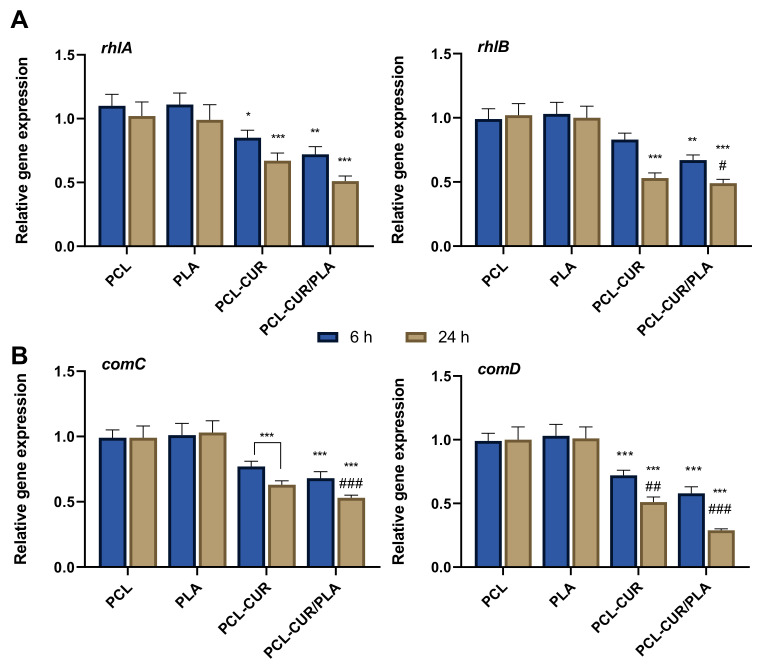
Evaluation of the effect of the CUR-loaded membranes on the expression of QS-related genes. (**A**) Relative RNA expression of *rhlA* and *rhlB* in PAO1; (**B**) relative RNA expression of *comC* and *comD* in *S. mutans*. Different gene expression levels were normalized to the level of 16sRNA gene transcripts. Gene expression levels of biofilm formed in the presence of PCL were used as control. Statistically significant variations: * *p* < 0.05 versus PCL and PLA; ** *p* < 0.01 versus PCL and PLA; *** *p* < 0.001 versus PCL and PLA; ^#^ *p* < 0.05 versus PCL-CUR; ^##^ *p* < 0.01 versus PCL-CUR; ^###^ *p* < 0.001 versus PCL-CUR.

As shown in Figure 6, both PCL-Cur and PCL-Cur/PLA significantly (*p* < 0.001) decreased the mRNA levels of all genes tested, compared to control (gene expression level in biofilm formed in the presence of PCL and PLA membranes). In particular, for PAO1, the relative expression levels of the virulence genes *rhlA*, and *rhlB* were significantly decreased by 0.85-, and 0.83-fold, respectively, after 6 h of incubation in the presence of PCL-Cur, while by 0.72- and 0.73-fold after 6 h of incubation in the presence of PCL-Cur/PLA. This reduction was more evident after 24 h of incubation, reaching a downregulation of 0.67- and 0.53-fold for PCL-Cur and 0.53- and 49-fold for PCL-Cur/PLA. The same behavior was observed for *S. mutans* biofilm, where the treatment with PCL-Cur and PCL-Cur/PLA for 6 h repressed the relative expression levels of *comC* and *comD* to 77% and 72% (PCL-Cur), and 37% and 49% (PCL-Cur/PLA), respectively, as compared to control values. After 24 h of incubation, only the relative expression level of com D significantly (*p* < 0.001) was downregulated, reaching a 29% decrease compared to control for PCL-CUR/PLA.

**Figure 7:**
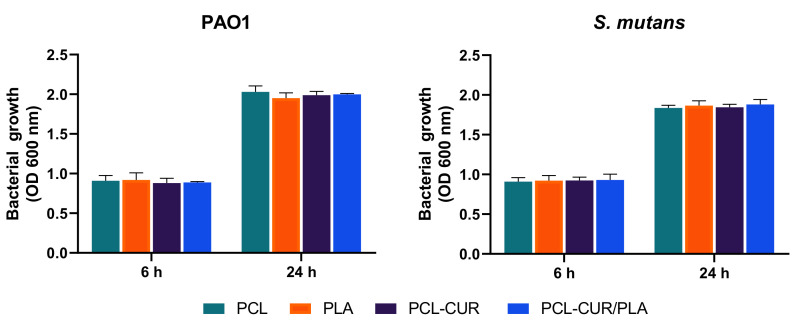
Antibacterial activity evaluated at 600 nm against *Pseudomonas aeruginosa* PAO1, and *Streptococcus mutans*. Bacterial growth in the presence of PCL and PLA were used as controls. For each sample, six different experiments were conducted, and the results expressed as the mean of the values obtained (mean ± SD).

Antimicrobial activity was then analyzed at 6 and 24 h in the presence of prepared membranes. As reported in Figure 7, the antibacterial evaluation demonstrated that the electrospun membranes did not have a noticeable effect on both the *Pseudomonas aeruginosa* PAO1 and the *Streptococcus mutans* growth curves.

The reported results demonstrated that the curcumin released from electrospun membranes is able to affect biofilm formation without interfering with bacterial growth.

### 2.6. Determination of DPPH Radical Scavenging Activity

Inflammation represents an adaptive physiological response to external and/or internal deleterious circumstances, including infection and tissue injuries. Extensive studies have suggested that continuous oxidative stress activates the inflammatory signaling cascade producing new free radicals that, in turn, lead to further oxidative stress, thus creating a cycle. This altered status can cause various chronic diseases such as cancer, atherosclerosis, Alzheimer’s disease, metabolic disorders, and so on [64,65]. Among the numerous phytochemicals, curcumin exhibited anti-inflammatory, antioxidative, and antitumor effects both on in vitro and in vivo models of human diseases [66]. The anti-inflammatory and antioxidative activity of curcumin is mostly related to the regulation of the NF-κB (nuclear factor k-light-chain-enhancer of activated B cells) pathway and the inhibition of the cyclooxygenase-2 (COX-2) [67]. Other studies reported the involvement of curcumin in the increase of reactive oxidative species (ROS) and glutathione production and inhibition of lipid peroxidation activity [68]. It has been suggested that the antioxidant activity of curcumin can be attributed either to the phenolic OH group or the CH_2_ group of the β-diketone moiety [69,70]. Indeed, the radical scavenging activity of curcumin is 100-fold stronger than that of vitamin E or C both in vitro and in vivo [71].

The ability of curcumin released from membranes to produces an antioxidant effect was evaluated by the DPPH radical scavenging assay, a simple and highly sensitive method to evaluate the free radical scavenging activity of antioxidants [72].

**Figure 8:**
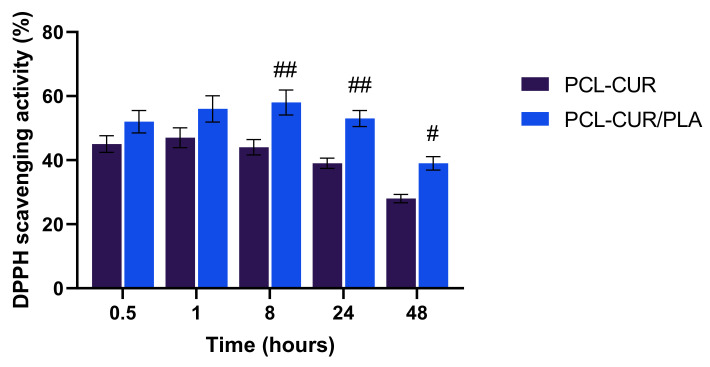
DPPH scavenging activity of Cur loading membranes. For each sample, six different experiments were conducted, and the results expressed as the mean of the values obtained (mean ± SD). Statistically significant variations: ^##^ *p* < 0.005 versus PCL-Cur; ^#^ *p* < 0.05 versus PCL-Cur.

As reported in Figure 8, a decrease in the concentration of DPPH radical was shown due to the scavenging ability of curcumin released from both PCL-Cur and PCL-Cur/PLA. However, the PCL-Cur/PLA coaxial membrane showed a significantly higher antioxidant activity with respect to PCL-Cur after 8, 24 and 48 h of immersion. No effect was observed in the presence of PLA-Cur and PLA-Cur/PCL membranes. Moreover, the results indicated that a longer immersion period (48 h) led to a reduction of antioxidant activity, likely due to decreasing stability of curcumin in alcohol solution.

### 2.7. Membranes Biocompatibility

**Figure 9:**
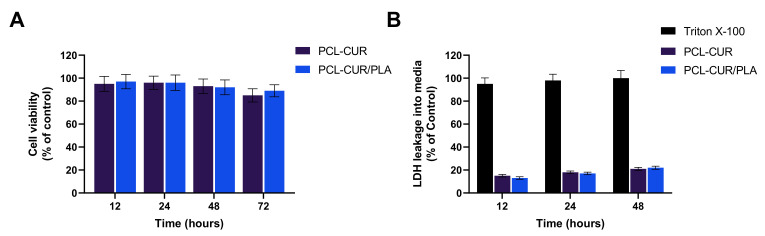
Cytotoxicity of curcumin released from Cur loading membranes tested via CCK-8 (**A**) and LDH (**B**) assays after 12, 24, 48, and 72 h of incubation. For each sample, six different experiments were conducted, and the results were expressed as the mean of the values obtained (mean ± SD).

To investigate the effect of curcumin extract on viability, human dermal fibroblasts (HDFs) were chosen as in vitro cell models. The results depicted in Figure 9 demonstrated that the extracts obtained in culture medium from all synthesized membranes did not elicit any cytotoxic effect. In particular, no influence on HDF metabolic activity was reported even after 72 h of incubation (Figure 9A). In addition, the low LDH level in the cell supernatant confirms the absence of cell membrane damage (Figure 9B).

Taken together, these results demonstrate that the fabricated membranes could be employed as scaffolds in tissue engineering regeneration to control the oxidative stress of damaged tissue, helping to accelerates the healing process.

## 3. Materials and Methods

### 3.1. Materials

Poly(ε-caprolactone) (PCL molecular weight of 80,000 Da) was purchased from Sigma Aldrich while poly (l-lactide-co-d,l-lactide) (PLA 4032 D-Mw = 160,000 g/mol) was purchased from NatureWorks (Minnetonka, MN, USA). Tetrahydrofuran (THF pure-CAS: 109-99-9), Ethanol (EtOH purity > 96%-CAS 64-17-5) and Phosphate Buffer Solution (PBS-pH = 7 ± 0.02-CAS: 7558-79-4) were purchased from Carlo Erba Reagents (Cornaredo-Milano). N,N-Dimethylformamide (DMF-CAS 68-12-2) and Curcumin (Cur) were purchased from Sigma Aldrich (Milan, Italy). Cell Counting Kit-8 (CCK-8) assay and Lactate dehydrogenase assay were from Roche Applied Science (Milan, Italy). Human dermal fibroblasts were purchased from the American Type Culture Collection ATCC (LGC Standards S.R.L., Sesto San Giovanni, Milan, Italy) and cultured in accordance with the manufacturer’s instructions. Fetal bovine serum (FBS), Dulbecco’s Modified Eagle’s Medium (DMEM), sodium pyruvate, L-glutamine, penicillin, and streptomycin were purchased from Hyclone (Milan, Italy).

### 3.2. Preparation of Curcumin-Loaded Membranes Using Electrospinning

The electrospinning membranes were prepared by dissolving PCL and PLA in a solvent mixture THF/DMF (50:50 *v/v*) at 12% *w*/*w*. Curcumin was added to PCL solution at the drug to polymer ratio of 0.1:9.9 (*w*/*w*) and mixed for 4 h at 40 °C using a temperature-controlled stirring plate (300 rpm) to obtain a homogenous solution. Coaxial electrospun membranes were obtained by using a coaxial nozzle (EM-CAX-Ime electrospinning). Two separate volumetric pumps were used to process the polymeric solutions, prepared as previously described. The curcumin-loaded coaxial nanofibrous mats were processed by coaxial electrospinning; the drug-loaded solution constitutes the inner core, while the no-loaded solution is the outer shell. Before performing the experiment, each solution was fed in a 5 mL syringe pump. The sets of electrospinning conditions were reported in Table 3 and optimized to produce nanofibrous mats without bead formation [44]. Temperature and relative humidity were fixed for all the experiments and equal to 25 °C and 35%, respectively. The produced composite nanofibrous mats were marked as PCL-Cur and PCL-Cur (core)/PLA (shell).

A climate-controlled electrospinning apparatus EC-CLI (IME Technologies, WG Waalre, The Netherlands) was used to produce fibrous membranes. The vertical setup was chosen to carry out the experiments. Core–shell nanofibers were obtained through a coaxial apparatus containing two concentric needles. The diameters of the inner and outer needles were 0.8 mm and 1.2 mm, respectively. The inner needle was 0.20 mm longer than the outer needle. For all the experiments, an aluminum collector was used to recover the electrospun nanofibers.

### 3.3. Morphological Analises

#### 3.3.1. Scanning Electron Microscopy (SEM)

SEM was carried out using a Quanta 200 F microscope (Thermo Fischer, Hillsboro, OR, USA) in high-vacuum mode. Before the analysis, electrospun membranes were covered with a thin film of gold using an Agar Automatic Sputter Coater (Mod. B7341, Stansted, UK) at 40 mA for 120 s prior to the analysis.

#### 3.3.2. Transmission Electron Microscopy (TEM)

TEM was performed on a FEI Tecnai 200 kV electron microscope (Thermo Fischer, Hillsboro, OR, USA) operating at 100 keV. The samples for the TEM observation were prepared by directly depositing the as-spun fibers onto the copper grids.

### 3.4. Structural Characterization of Electrospinning Nanofibers

#### 3.4.1. Thermogravimetric Analyses (TGA)

TGA were carried out in an air atmosphere with a Mettler TC-10 thermobalance (Mettler Toledo GmbH, Greifensee, Switzerland) from 25 °C to 800 °C at a heating rate of 10 °C/min.

#### 3.4.2. Mechanical Properties

Mechanical properties were evaluated, in tensile mode, at room temperature using a dynamometric apparatus INSTRON 4301 (ITW Test and Measurement Italia S.r.l., Pianezza, Italy). Experiments were conducted at room temperature with a deformation rate of 5 mm/min. Elastic modulus was evaluated in the deformation range of 0.1%. Data were averaged on five samples.

### 3.5. Curcumin Entrapment Efficiency

Square pieces of membranes (ca. 2 cm^2^) were weighted and dissolved in methanol (1:5 *w*/*v*) for 60 min and curcumin concentration was measured as reported above by HPLC. The curcumin loading efficiency (LE) was calculated as percentage respect to the loaded drug as follows:

$$\mathrm{LE}\;\left(\%\right)=\frac{\mathrm{curcumin}\;\mathrm{concentration}}{\mathrm{curcumin}\;\mathrm{concentration}\;\mathrm{initially}\;\mathrm{added}\;\mathrm{in}\;\mathrm{the}\;\mathrm{polymer}\;\mathrm{solution}}\times 100$$

### 3.6. In Vitro Release Kinetic Measurement

The release of the curcumin from the membranes was determined as early described with some modifications [73]. Briefly, square pieces of membranes (ca. 2 cm^2^) were weighed and placed into individual amber vials. Considering the very low solubility of the curcumin in aqueous solutions, the release kinetic was performed at 37 °C in PBS/EtOH (90:10 *v/v*). At predetermined time intervals (every hour for 6 h, then at 12, 24, 48 and 72 h), supernatants were withdrawn, and the same amount of fresh solution was added back to the release medium. The curcumin concentration was measured using HPLC-UV with a linear elution gradient consisting of mobile phase A (0.1% acetic acid), B (Acetonitrile), and C (Methanol). The detection wavelength was set at 420 nm and Curcumin quantitation was based on a standard curve in PBS/EtOH (90:10 *v/v*). System control and data acquisition were performed using ChemStation software (Agilent Technologies). The results were presented in terms of cumulative release (percentage with respect to the loaded drug) as a function of time.

### 3.7. Antioxidant Activity

Antioxidant activity, as the free radical-scavenging ability of curcumin-loaded nanofibers, was examined using 1,1-dipheny-l-2-picryl hydrazyl (DPPH) assay as reported by Amrati et al. with slight modifications [74]. Samples of square pieces of membranes (ca. 1.5 × 2 cm) were dissolved in PBS/EtOH (90:10 *v/v*) for 0.5, 1, 8, 24 and 48 h and then ultrasonicated for 15 min. The dilution of the solutions was done in PBS/EtOH (90:10 *v/v*). Aliquots (500 μL) of those solutions were added to 2 mL of DPPH methanolic solution (60 μM) and kept in the dark at 37 °C for 1 h. The absorbance of the samples was determined at 517 nm using a microplate reader (Cytation 3, ASHI). A methanolic solution of free curcumin and polymers was used as a control. The percentage of inhibition of DPPH was calculated as follows:

DPPH scavenging effect (%) = [(A_1_ − A_0_)/A_1_] × 100

where A_1_ was the absorbance of the control (DPPH solution without sample) at 517; A_0_ was the absorbance at 517 of the sample at different concentrations with DPPH. The antioxidant activity was expressed as % with respect to free curcumin.

### 3.8. Citotoxicity

#### 3.8.1. Cell Proliferation Assay

Indirect cytotoxicity evaluation of curcumin-loaded membranes was conducted according to ISO 10993-5 standard test method as reported by Conte et al. with slight modifications [75]. Samples in a circular shape (1.5 cm in diameter) were exposed to UV radiation for 30 min for sterilization. Then, the samples were immersed in 10 *v/v* % serum containing DMEM medium for 24 h at 37 °C to produce the sample extraction. Human dermal fibroblasts (HDFs) were cultured in 96-well tissue-culture polystyrene plate (TCPS) at 2 × 10^3^ cells/well in serum-containing DMEM for 16 h to allow cell attachment. After that, the medium was replaced with an extraction medium and HDFs were incubated for a further 12, 24 and 48 h. At the end of the incubation period, all CCK-8 solutions were added to each well, and the plate was then incubated under cell culture conditions for 1–4 h. The optical density of formazan salt at 450 nm was measured using a Citation 3 Cell Imaging Multi-Mode microplate reader (ASHI, Milan, Italy). The cytocompatibility of the membranes was expressed as a percentage relative to the control and calculated as:

Cytocompatibility (%) = (OD sample/OD control) × 100

where OD sample is the optical density of cells treated with curcumin extract and OD control is the optical density of untreated cells.

#### 3.8.2. LDH Release Assay

Lactate dehydrogenase (LDH) release measurements were based on the measurement of lactate LDH released into the growth media when the integrity of the cell membrane is lost. For this assay, HDF cells were treated with extracts in the same way as described above. At the end of the incubation time, 100 μL of the culture supernatants were collected to a well, and LDH activity was detected at 450 nm as reported by manufacturing protocol. As a positive control, cells were completely lysed with Triton X-100, according to Calarco et al. [76].

### 3.9. Antimicrobial Activity

#### 3.9.1. Bacterial Strains and Culture Conditions

*Pseudomonas aeruginosa* PAO1 (ATCC^®^ BAA-47™) and *Streptococcus mutans* (ATCC^®^ 25175) were purchased from ATCC, and cultured following the ATCC’s guidelines.

#### 3.9.2. Antibacterial Activity

The capability of the curcumin-loaded membranes to inhibit bacterial growth was determined by monitoring the optical density (OD) at 600 nm of bacterial suspensions cultured in the presence of both PCL-Cur and coaxial PCL-Cur/PLA. All mats were cut of similar dimension, sterilized by UV radiation for 15 min at each side, and finally placed in a 12-well plate in the presence of 500 µL liquid broth as previously described [77]. Briefly, bacteria were inoculated approximately at 1 × 10^7^ CFU/mL and incubated at 37 °C and 200 rpm in a microplate reader (Cytation 3). As a control, the growth curve was obtained in the presence of PCL mat. At scheduled times (6 h or 24 h), the optical density (OD) at 600 nm was recorded.

#### 3.9.3. Biofilm Analysis

To investigate the ability of the curcumin-loaded membranes to inhibit the biofilm formation, a similar amount of PCL-Cur, and PCL-Cur/PLA were sterilized by UV radiation for 15 min at each side, placed in a 48-well polystyrene plate, and biofilm developed as described by Di Salle et al. with some modifications [78]. Briefly, 750 µL of liquid medium broth containing 1 × 10^7^ CFU/mL of *S. mutans* or PAO1 were added and the cultures were incubated statically at 37 °C in a humid atmosphere. PLA or PCL mats incubated in liquid medium broth were used as a negative control, while 750 µL of PAO1 (1 × 10^7^ CFU/mL) or *S*. *mutans* (1 × 10^7^ CFU/mL) were used as positive controls.

The surface-adhered biofilm was quantified after 6, 12, and 24 h by the Crystal Violet (CV) assay. Each well was washed gently with sterile phosphate-buffered saline (PBS), and air-dried for 30 min. Then, a solution of 0.1% *w/v* Crystal Violet was added to each well. After 30 min, excess solution was removed, and any extra stain was removed by washing with PBS. The stained biofilms were solubilized in 96% ethanol and quantified by measuring the optical density (OD) at 570 nm using a microplate reader (Cytation 3, AHSI, Milan, Italy). Measurements were carried out in triplicate for each membrane.

#### 3.9.4. Quorum Sensing (QS) Interfering

To determine the ability of the membranes to interfere with the quorum-sensing mechanism underlying the biofilm maturation process, the mRNA level of *rhlAB* genes for PAO1 and of *comCD* genes for *S. mutans* was quantified by real-time PCR (qRT-PCR). PAO1 and *S. mutans* biofilms were developed in the presence of synthesized membranes for 6 or 24 h, as previously described in a 48-well polystyrene plate. The bacterial pellet was then collected by centrifugation at 13,000× *g* for 10 min, and total RNA was extracted using TRIzol reagent (Invitrogen, Italy) as previously described [79]. Briefly, 0.2 μg of total RNA was retrotranscripted using AMV Reverse Transcriptase and random hexamers according to the provider’s instruction (Promega, Milan, Italy). The resulting mixture was amplified by qRT-PCR using specific primers based on the previous literature as listed in Table 4.

qPCR and data collection were performed on 7900HT Fast Real-time PCR System (Applied Biosystems, Milan, Italy). The reactions were performed according to the manufacturer’s instructions using SYBR Green PCR Master Mix (Invitrogen, Italy). All reactions were run in triplicate, normalized to the housekeeping gene (*16SrRNA*), and the results expressed as mean ±SD. The comparative cycle threshold (2−ΔΔCt) method was used to determine the relative quantification.

### 3.10. Statistical Analysis

Results were expressed as mean ±  standard deviation (SD). Student’s *t*-test was used for the curcumin release. For biochemical, antimicrobial investigations assay, and quantitative real-time PCR one-way analysis of variance (ANOVA) with Tukey’s post-hoc test were used for statistical comparison. The difference was regarded as statistically significant when *p* < 0.05. All the data were analyzed with the GraphPad Prism version 6.01 statistical software package (GraphPad, San Diego, CA, USA).

## 4. Conclusions

In this study, electrospinning was used to produce nanofibrous membranes with the ability to release curcumin in a continuous and sustainable mode, eliciting both antibacterial and antibiofilm activity on *Pseudomonas aeruginosa* PAO1 and *Streptococcus mutans*—two opportunistic bacteria frequently associated with human infections. In particular, we observed a more important effect, especially after 12 h of incubation, with the coaxial respect to the single-needle electrospun membrane, showing a reduction of 23 ± 5%, 33 ± 4% in PAO1 and *S. mutans* biofilm formation, respectively. Our data also demonstrated the ability of CUR-loading membranes to inhibit signal-based biofilm formation, lowering the expression of several genes involved in the quorum-sensing mechanism that led to biofilm maturation.

Moreover, the presented results demonstrated good biocompatibility of both PCL-Cur, and PCL-Cur/PLA and a good antioxidant capacity already after 30 min of incubation.

Taken together, these results demonstrate that the fabricated membranes could be employed as scaffolds in tissue engineering regeneration to control the oxidative stress of damaged tissue, thus helping to accelerates the healing process.

## Figures and Tables

**Table 1:** Onset temperatures, endset temperatures and residue contents of electrospun membranes.

	PCL-Cur	PCL-Cur/PLA	PLA
Residue (%)	1.89	4.70	2.98
Onset (°C)	372	260	315
Endset (°C)	425	395	374

**Table 2:** Mechanical properties of electrospun membranes.

	PCL-Cur	PCL-Cur/PLA
E (MPa)	6.1 ± 0.5	48 ± 25
σ_break_ (MPa)	2.19 ± 1.1	1.31 ± 0.3
ε _break_ (mm/mm%)	139 ± 3.5	46 ± 11

**Table 3:** Processing parameters of fibrous membranes fabricated by electrospinning.

Sample	Polymer Concentration (% *w*/*w*)	Voltage (kV)	Distance (cm)	Flow Rate (mL/h)
PCL-Cur	12	17.5	18	0.5
Core: PCL-Cur Shell: PLA	Core: 12 Shell: 12	24	25	Core: 0.5 Shell: 0.7

**Table 4:** QPCR primers.

Gene	Forward Primer (5′—3′)	Reverse Primer (5′—3′)	Ref.
*rhlA*	AGCTGGGACGAATACACCA	GACTCCAGGTCGAGGAAATG	[80]
*rhlB*	GAGCGACGAACTGACCTACC	GTTGAACTTGGGGTGTACCG	[80]
*comC*	GACTTTAAAGAAATTAAGACTG	AAGCTTGTGTAAAACTTCTGT	[81]
*comD*	CTCTGATTGACCATTCTTCTGG	CATTCTGAGTTTATGCCCCTC	[81]
*16SrRNA*	CCTACGGGAGGCAGCAGTAG	CAACAGAGCTTTACGATCCGAAA	[82]
*16SrRNA*	CAAAACTACTGAGCTAGAGTACG	TAAGATCTCAAGGATCCCAACGGCT	[83]

## Data Availability

Not applicable.

## Sample Availability

Not available.
